# Integrating migration into cancer geography: spatial clustering of five digestive cancer burden in China

**DOI:** 10.3389/fpubh.2026.1791882

**Published:** 2026-05-22

**Authors:** Guijin Li, Liangfang Xue, Shuxiu Hao, Linlin Du, Huixin Sun, Yu Zhang, Qiao Lu, Ming Liu, Yaru Wang, Yuehui Jia, Chen Feng, Tong Wang, Qi Li

**Affiliations:** 1 Chinese Center for Endemic Disease Control, Harbin Medical University, Harbin, Heilongjiang, China; 2 NHC Key Laboratory of Etiology and Epidemiology, Harbin Medical University, Harbin, Heilongjiang, China; 3 Joint Key Laboratory of Endemic Diseases (Harbin Medical University, Guizhou Medical University, Xi’an Jiaotong University), Harbin, Heilongjiang, China; 4 Department of Radiation Oncology, Harbin Medical University Cancer Hospital, Harbin, Heilongjiang, China; 5 Department of Biostatistics, School of Public Health, Qiqihar Medical University, Qiqihar, Heilongjiang, China; 6 Cancer Center of Heilongjiang Province, Harbin Medical University, Harbin, Heilongjiang, China; 7 Environmental Health Department, Liaoyuan Center for Disease Control and Prevention, Liaoyuan, China

**Keywords:** county-level analysis, digestive cancers, migration-adjusted burdens, population migration, spatial clustering

## Abstract

**Introduction:**

China’s digestive cancer landscape presents a dual challenge, the significant geographic disparity and the distorting effect of large-scale internal migration on burden estimation. The household-registered population (HRP)-based surveillance system does not fully capture the impact of large-scale internal migration, and county-level spatial evidence that accounts for migration remains limited. This study aimed to characterize the spatial distribution of the migration-adjusted burden of five digestive cancers in China, assess spatial autocorrelation, and identify county-level high-high and low-low clusters.

**Methods:**

This spatial epidemiological study used previously developed migration-adjusted county-level incidence and mortality estimates for five digestive cancers in mainland China, derived using Bayesian INLA-SPDE modeling based on quality-controlled data from 487 cancer registries covering 28.6% of the national population. The spatial distribution was visualized using thematic mapping, and Global and Local Moran’s *I* analyses were conducted to assess spatial autocorrelation and identify significant spatial clusters.

**Results:**

Spatial description revealed geographic patterns in cancer incidence and mortality, closely corresponded to clusters identified by spatial autocorrelation analysis. Global Moran’s *I* analysis confirmed significant positive spatial autocorrelation in the migration-adjusted burden of all five cancers nationwide (*p* < 0.001). Distinct geographic patterns of HH clusters were evident across cancers: liver cancer in the southeastern coast, colorectal cancer in the eastern coast and northeast, gastric cancer in the east and northwest, esophageal cancer in central-eastern regions, and pancreatic cancer in the northeast and eastern coast. LL clusters were mainly in western, central-western, and southwestern China.

**Conclusion:**

This study identified significant spatial clustering in the migration-adjusted burden of the five major digestive cancers in China. High-burden areas were mainly concentrated in eastern coastal, northeastern, and parts of central-eastern China, whereas lower-burden areas were more common in western and parts of southwestern China. These findings highlight the importance of incorporating population mobility into digestive cancer burden assessment and provide evidence to support region-specific cancer prevention, control prioritization, and more dynamic surveillance in China.

## 1 Introduction

Digestive cancers comprise a heterogeneous group of malignancies arising from multiple anatomical sites and collectively account for a substantial proportion of the global cancer burden. In 2022, new cases of digestive cancers in China accounted for 7.9% of all cancer cases worldwide and 33.4% of global digestive cancer cases, deaths from these cancers represented 11.4% of all cancer deaths worldwide and 34.4% of deaths from digestive cancers (1, 2). Among these malignancies, liver, colorectal, gastric, esophageal, and pancreatic cancers, which are hereafter collectively referred to as the five digestive cancers, represent the digestive cancer types with the highest incidence in China, with incidences in 2022 of 26.0, 36.6, 25.4, 15.9, and 8.4 per 100,000 population, respectively (3). Studies have shown that digestive cancer burden exhibits substantial geographic heterogeneity worldwide, and that spatial epidemiological approaches can help identify geographic clustering patterns and inform targeted interventions (4–6). Consistent with this broader evidence, previous studies have consistently documented substantial spatial heterogeneity in digestive cancer burden in China, but they have primarily examined individual cancer types, specific provinces or regions, or burden estimates based on household-registered population (HRP) (7–10). Importantly, in the context of large-scale internal migration in China, reliance on HRP-based data may further distort the spatial assessment of cancer burden. Data from the Seventh National Population Census indicate that more than 370 million individuals are internal migrants in China in 2020 (11), leading to fundamental discrepancies between HRP, which refers to the population recorded in the household registration system, and resident population (RP), which refers to the population actually living in a given area. However, the existing cancer registry system is based on HRP and does not adequately account for population mobility (12, 13), thereby introducing substantial selection bias: cancer burden may be underestimated in immigrant provinces and overestimated in emigrant provinces. Although the impact of population mobility on cancer burden has been increasingly recognized, no district/county-level spatial analysis of digestive cancers has been conducted using migration-adjusted burden data. We have previously developed Bayesian INLA-SPDE models using data from 487 cancer registries to estimate county-level burden of five digestive cancers adjusted for population mobility. Building on this dataset, we conducted a spatial epidemiological analysis to systematically characterize the spatial distribution of the migration-adjusted burden of major digestive cancers in China, identify and localize spatial HH and LL clusters at the district/county level, and provide spatial epidemiological evidence for digestive cancer prevention and control strategies.

## 2 Materials and methods

### 2.1 Study design

**Figure 1:**
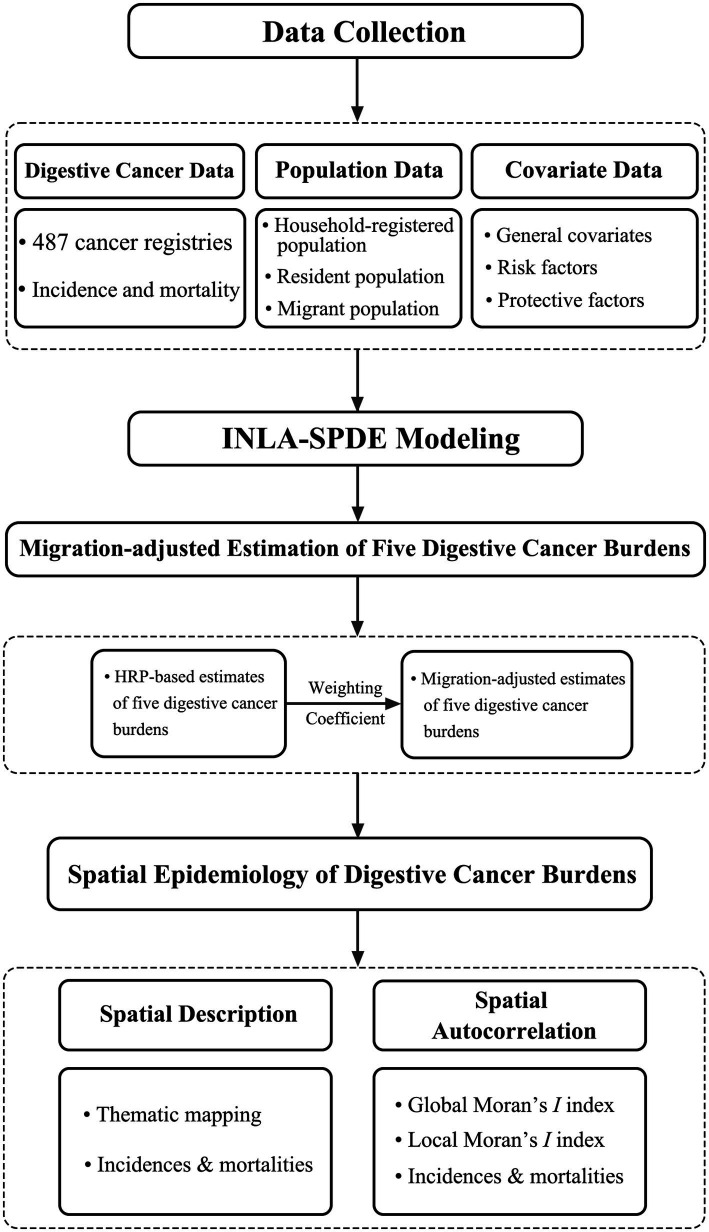
Study flow diagram.

This is a spatial epidemiological study that utilizes migration-adjusted county-level incidence and mortality for five digestive cancers to systematically characterize the spatial distribution of cancer burden across mainland China and to identify spatial clusters. The overall workflow of the study is illustrated in Figure 1.

### 2.2 Source of data

The 2,845 migration-adjusted district/county-level incidence and mortality estimates used in this study were derived from our previous work for five digestive cancers, namely liver, colorectal, gastric, esophageal, and pancreatic cancers, defined according to the International Statistical Classification of Diseases and Related Health Problems, 10th Revision (ICD-10), with codes C22, C18-C20, C16, C15, and C25, respectively (14). In 2016, a total of 682 cancer registries were available in mainland China, of which 487 (71.4%) from 31 provinces, covering 687 districts and counties, met the data quality requirements for cancer registration established by the International Agency for Research on Cancer/International Association of Cancer Registries (IARC/IACR) and Cancer Incidence in Five Continents, Volume 11 (15). The included registries covered 28.6% of China’s HRP. Using data from these 487 eligible registries, we applied Bayesian INLA-SPDE spatial models to estimate the incidence and mortality of the five digestive cancers at the district/county level (16–18). Registry-level cancer counts were modeled with the HRP as an offset term, and covariate effects together with spatial random effects were incorporated to capture geographic dependence and generate estimates for districts/counties without cancer registries. The analysis code used in this study is available at https://github.com/keyanyuan2021/INLA-SPDE. To characterize population mobility, net migrant population was defined as RP minus HRP, and interprovincial migration patterns were quantified using data from the 2016 China Migrant Population Dynamic Survey (19). The final estimates were further adjusted by incorporating the provincial composition and age structure of the migrant population to obtain migration-adjusted incidences and mortalities at district/county level (15, 20).

### 2.3 Spatial description

By creating thematic maps, we visualized the migration-adjusted incidence and mortality of five digestive cancers to provide an intuitive representation of their spatial distribution and geographic variation across mainland China.

### 2.4 Spatial autocorrelation analysis

To assess whether significant spatial clustering (aggregation or dispersion) exists in the migration-adjusted incidences and mortalities of five digestive cancers across mainland China at the global level, we employed Global Moran’s *I* analysis. Its mathematical expression is as follows:

$$I=\frac{n}{\sum_{i=1}^{n}{\left(y_{i}-\bar{y}\right)}^{2}}\;\;.\;\;\frac{\sum_{i=1}^{n}\sum_{j=1}^{n}w_{ij}\left(y_{i}-\bar{y}\right)\left(y_{j}-\bar{y}\right)}{\sum_{i=1}^{n}\sum_{j=1}^{n}w_{ij}}$$

*n* denotes the number of spatial units; *y_i_* represents the observed value of the unit *i*; *ӯ* indicates the mean of all observed values; and *w_ij_* refers to the spatial weight matrix, which defines the spatial relationship between units *I* and *j*. In this study, a first-order Queen contiguity-based spatial weights matrix was used, in which districts/counties sharing a common boundary or vertex were defined as neighbors. *I* ranges from −1 to 1. *I* > 0 indicates positive spatial autocorrelation; *I* < 0 indicates negative spatial autocorrelation; and *I* ≈ 0 suggests a random spatial pattern (21).

To identify the contribution of each spatial unit to the global autocorrelation and to locate areas with unusual rates of five digestive cancers’ incidence and mortality, Local Moran’s *I* analysis was performed. Its mathematical formulation is as follows:

$$I_{i}=\frac{\left(y_{i}-\bar{y}\right)}{S^{2}}\overset{n}{\underset{j=1}{\sum }}w_{ij}\left(y_{i}-\bar{y}\right)$$

$$S^{2}=\frac{\sum_{j=1}^{n}{\left(y_{i}-\bar{y}\right)}^{2}}{n}$$

The symbols in the formula carry the same meanings as in the Global Moran’s *I*. Local Moran’s *I* yields four types of outcomes. Among statistically significant units (*p* < 0.05), HH clusters indicate that unit i and its neighboring units both have values above the mean, representing hotspot areas; LL clusters indicate that unit i and its neighbors both have values below the mean, representing coldspot areas. HL and LH clusters represent two types of spatial outliers, with the former referring to a high-value unit surrounded by low-value neighbors, and the latter referring to a low-value unit surrounded by high-value neighbors (15, 20, 21). Notably, the local clustering results were interpreted with due caution, as no further false discovery rate (FDR) correction was introduced in the present analysis (22–24).

### 2.5 Statistical analysis

We used SPSS 17.0 for data organization and cleaning, R 4.3.2 for data preprocessing and model construction, and ArcGIS 9.0 for thematic mapping and spatial autocorrelation analysis.

## 3 Results

### 3.1 Spatial distribution of five digestive cancer incidences

**Figure 2:**
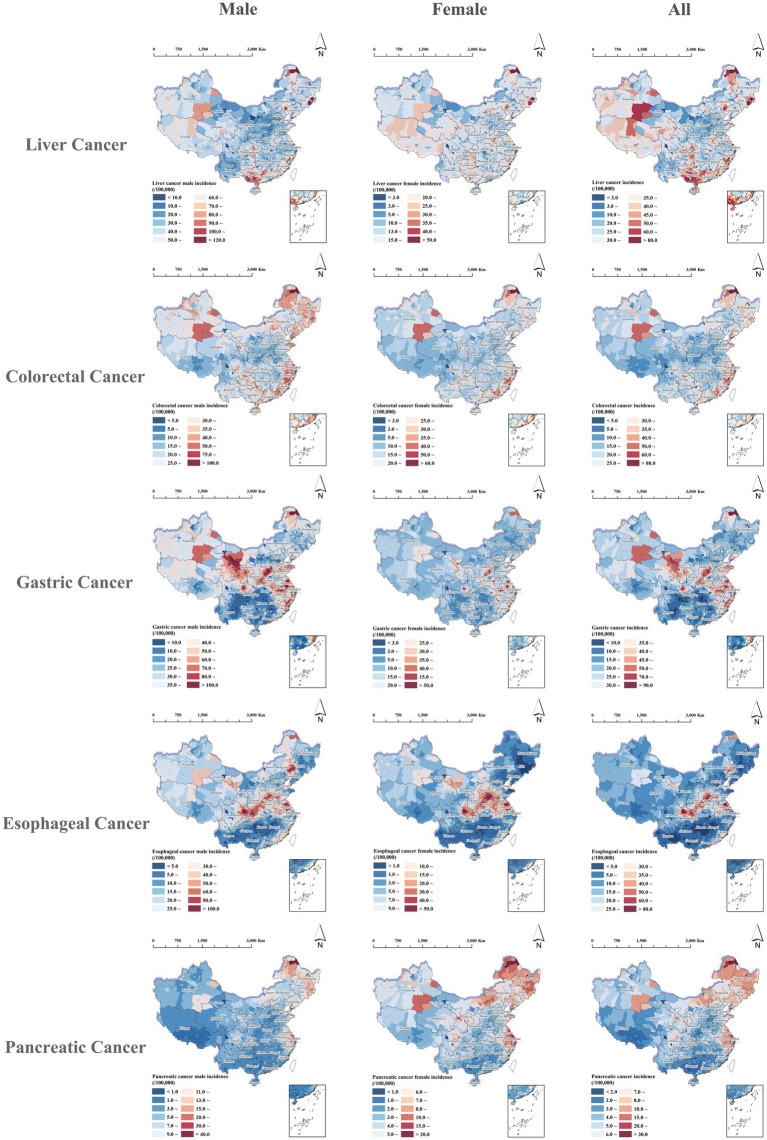
Spatial distribution of migration-adjusted incidences for five digestive cancers in China, stratified by sex.

The migration-adjusted incidence of the five major digestive cancers in China showed highly consistent spatial patterns across males, females, and the overall population, with pronounced heterogeneity nationwide. Overall, the burden tended to be higher in eastern coastal, northeastern, and parts of central-eastern China, whereas western and parts of southwestern China generally exhibited lower burden, although notable cancer-specific differences were also observed. Specifically, liver cancer showed a predominantly southern clustering pattern, with relatively high incidence in southern provinces such as Guangdong, Guangxi, and Fujian, contrasting with lower rates in western provinces like Qinghai, Xizang, and Xinjiang. Colorectal cancer was concentrated particularly in eastern coastal and northeastern provinces such as Jiangsu, Heilongjiang, and Jilin, while remaining lower in southwestern and most northwestern areas. Gastric cancer displayed a marked east–west contrast, with higher incidence in eastern provinces such as Jiangsu and Anhui as well as northwestern regions including Gansu and Xinjiang, compared to lower levels in southern and parts of southwestern provinces. Esophageal cancer showed a central-to-eastern concentration pattern, with relatively high incidence in provinces such as Shaanxi, Shanxi, Henan, and Jiangsu, in contrast to lower incidence along the southern coast and southwestern provinces. Pancreatic cancer, generally low nationwide, showed localized high-incidence areas in eastern and northeastern provinces including Jiangsu, Jilin, and Heilongjiang, whereas most counties across southern, western, and southwestern China remained at relatively low levels, as shown in Figure 2.

### 3.2 Spatial distribution of five digestive cancer mortalities

**Figure 3:**
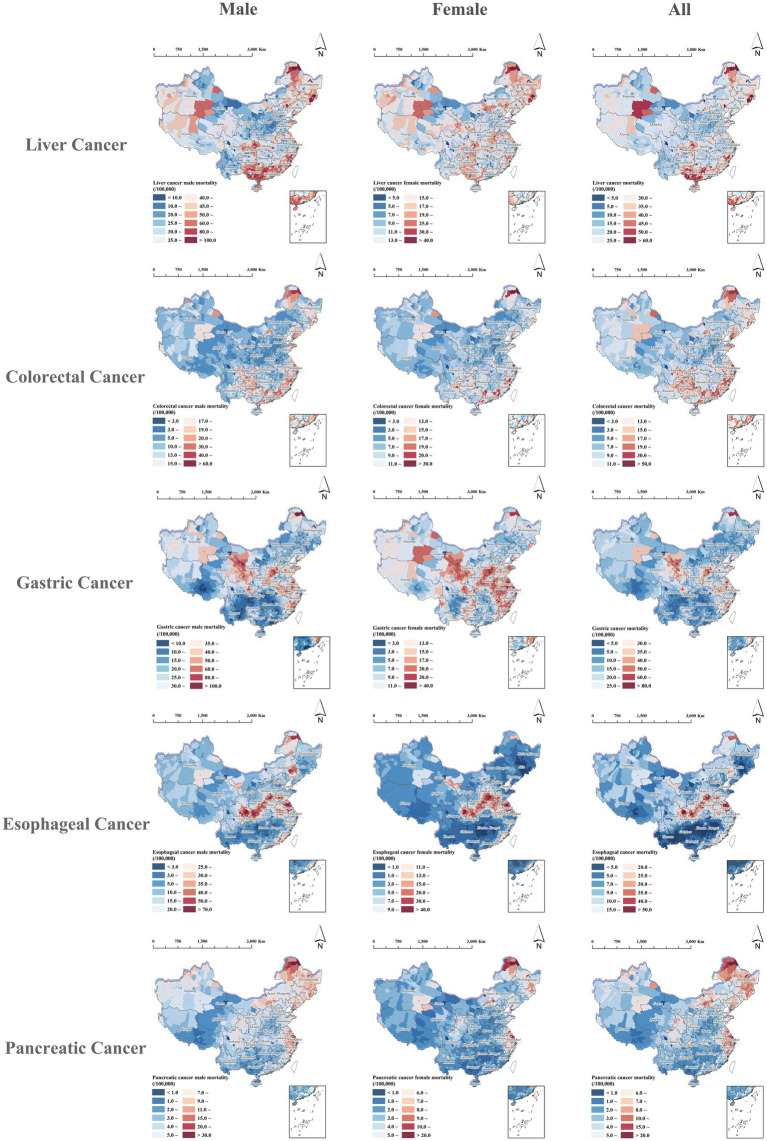
Spatial distribution of migration-adjusted mortalities for five digestive cancers in China, stratified by sex.

Similar to the incidence patterns, the migration-adjusted mortality of the five digestive cancers in China also showed highly consistent spatial patterns across males, females, and the overall population, with pronounced heterogeneity nationwide. Overall, the mortality burden tended to be higher in eastern coastal, northeastern, and parts of central-eastern China, whereas western and parts of southwestern China generally exhibited lower burden, although notable cancer-specific differences were also observed. Liver cancer showed a predominantly southern clustering pattern, with higher mortality in Guangdong, Guangxi, and Fujian and relatively elevated levels also observed in parts of northeastern China, whereas western and southwestern provinces including Qinghai, Yunnan, and Inner Mongolia showed relatively lower mortalities. Colorectal cancer was concentrated in eastern coastal and northeastern China, particularly in provinces such as Jiangsu, Zhejiang, Heilongjiang, and Jilin, in contrast to lower levels in southwestern and most northwestern provinces. Gastric cancer mortality mirrored its incidence, showing a clear east–west divide, with high-mortality areas mainly in eastern provinces such as Jiangsu and Anhui as well as in northwestern regions including Gansu and Xinjiang, while southern and some southwestern provinces remained at lower levels. Esophageal cancer showed relatively high mortality in central-to-eastern provinces such as Shaanxi, Shanxi, Henan, and Jiangsu, compared to lower mortality along the southern coast and in southwestern provinces. Pancreatic cancer mortality, though generally lower nationwide, was notably higher in eastern and northeastern provinces such as Jiangsu, Jilin, and Heilongjiang, while central, western, and southwestern counties mostly exhibited low mortality, as shown in Figure 3.

### 3.3 Global spatial autocorrelation analysis of five digestive cancer burden

**Figure 4:**
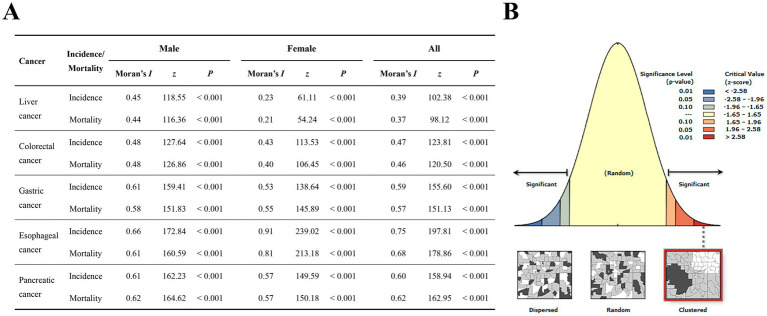
Global spatial autocorrelation of migration-adjusted incidence and mortality for five digestive cancers in China. **(A)** Global Moran’s I and significance tests for incidence and mortality by sex across five digestive cancers; **(B)** Schematic diagram of spatial distribution patterns and corresponding critical values for significance levels.

The results of the global spatial autocorrelation analysis are shown in Figure 4. For migration-adjusted incidence and mortality of five digestive cancers in China, the global Moran’s *I* values were positive across all cancer types and sexes, their corresponding *p* values all < 0.001, indicating significant positive spatial autocorrelation. Local spatial autocorrelation analysis of major digestive cancer incidences.

**Figure 5:**
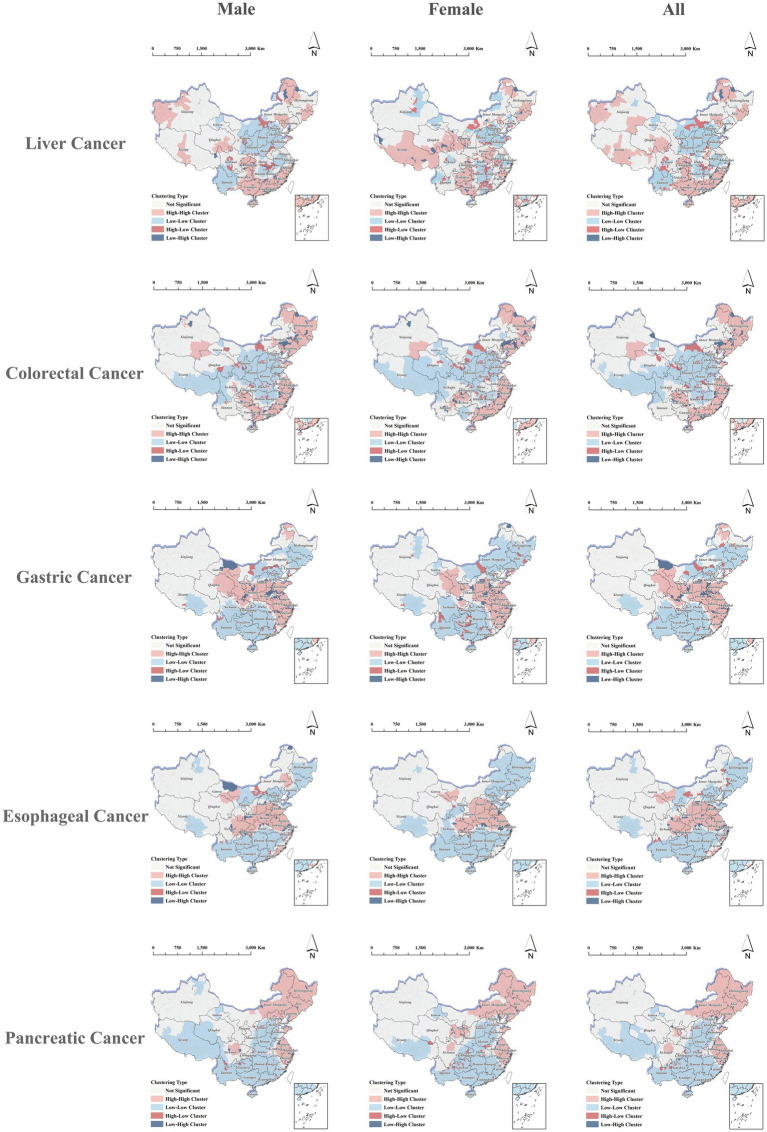
Local indicators of spatial association (LISA) clusters for migration-adjusted incidence of five digestive cancers in China, stratified by sex.

This study employed local spatial autocorrelation analysis to identify county-level HH clusters of migration-adjusted incidence for five digestive cancers. The spatial distribution of these HH clusters varied significantly by cancer type, whereas LL clusters were predominantly located in western and parts of central-western provinces. The spatial patterns of clusters identified in males, females, and the overall population were largely consistent, with local differences mainly attributable to the classification of individual counties. Specifically, HH clusters for liver cancer were mainly observed in southern coastal provinces such as Fujian, Guangdong, Guangxi, and Hainan, with additional clusters in certain counties of Xinjiang and Xizang, while LL clusters were predominantly located in central provinces such as Shaanxi and Shanxi, as well as Yunnan. HH clusters of colorectal cancer were primarily located in eastern coastal provinces such as Shanghai and Zhejiang, as well as northeastern provinces including Heilongjiang and Liaoning, with some HH clusters also observed in parts of Guangdong, Guangxi, and Guizhou; LL clusters were located across southwestern and northwestern provinces. For gastric cancer, HH clusters were mainly distributed in eastern coastal provinces such as Jiangsu and in northwestern provinces including Gansu and Qinghai, whereas LL clusters were primarily found in southern and southwestern provinces. HH clusters of esophageal cancer were mainly located in central and eastern provinces, including Henan, Jiangsu, and Anhui, while LL clusters were predominantly located in southern coastal and southwestern provinces. For pancreatic cancer, HH clusters were mainly observed in northeastern provinces such as Heilongjiang, Jilin, and Liaoning, as well as in eastern coastal provinces including Shanghai and Zhejiang, whereas LL clusters were mainly located in central, southern, and southwestern provinces. The provincial proportions of counties classified as HH clusters for each cancer type are presented in Supplementary Table S1, and the corresponding spatial distributions are shown in Figure 5.

### 3.4 Local spatial autocorrelation analysis of five digestive cancer mortalities

**Figure 6:**
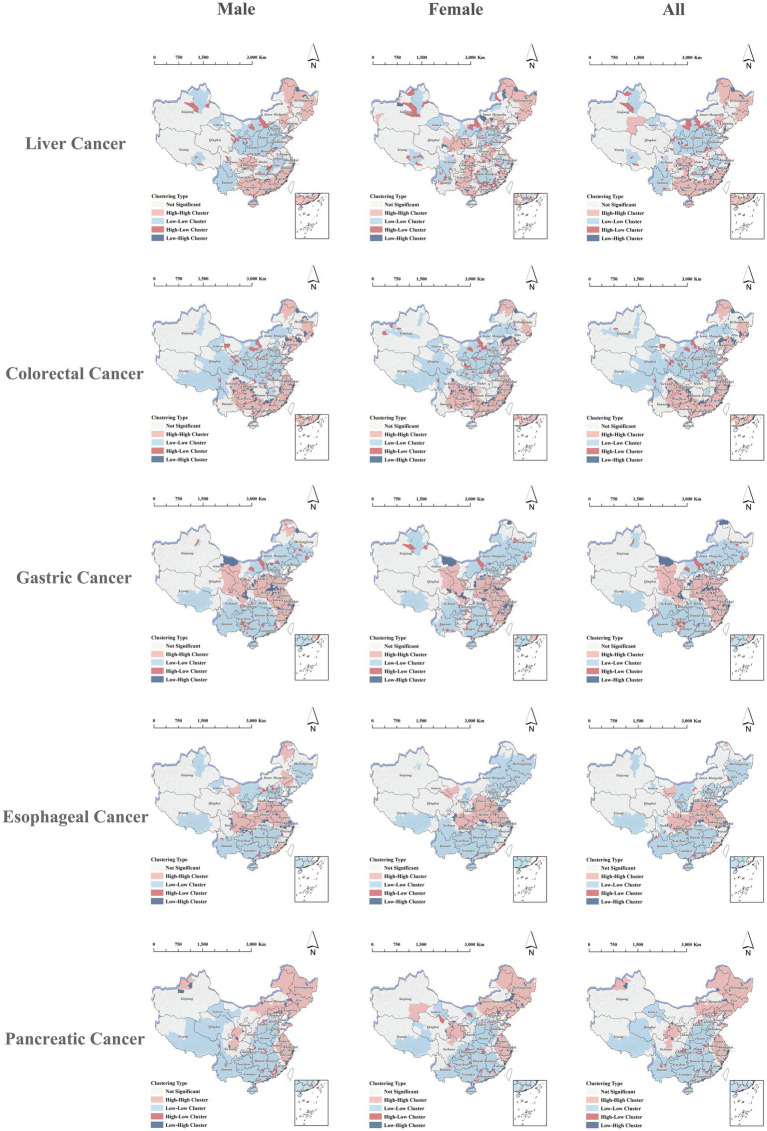
Local indicators of spatial association (LISA) clusters for migration-adjusted mortality of five digestive cancers in China, stratified by sex.

The local spatial autocorrelation analysis of mortality from five digestive cancers revealed marked variation in the spatial distribution of HH clusters across cancer types at the district/county level, with generally consistent clustering patterns observed among males, females, and the overall population. Specifically, HH clusters for liver cancer mortality were mainly concentrated in southeastern coastal provinces such as Fujian, Guangdong, and Guangxi, while LL clusters were predominantly distributed across central and northern provinces including Shanxi, Shaanxi, and Inner Mongolia, as well as southern provinces such as Yunnan and Sichuan. For colorectal cancer mortality, HH clusters were primarily observed in eastern and southern provinces including Zhejiang, Fujian, and Guizhou, as well as in northeastern provinces such as Liaoning, with LL clusters mainly located in central and southwestern provinces including Shaanxi, Shanxi, Qinghai, and Xizang. Gastric cancer mortality showed HH clusters in eastern and central provinces such as Jiangsu and Henan, with additional clusters in parts of western provinces including Gansu, while LL clusters were concentrated in southern and northeastern provinces. Esophageal cancer mortality exhibited a similar but geographically more restricted clustering pattern compared to gastric cancer, with HH clusters likewise concentrated in central and eastern provinces and LL clusters distributed across southern and northeastern provinces. For pancreatic cancer, HH clusters were mainly found in eastern coastal provinces such as Shanghai, Jiangsu, and Zhejiang, and in northeastern provinces including Heilongjiang and Jilin, whereas LL clusters were predominantly located in parts of central, southern, and southwestern provinces. The provincial proportions of districts/counties classified as HH clusters for each cancer type are presented in Supplementary Table S2, and the corresponding spatial distributions are shown in Figure 6.

## 4 Discussion

Against the background of large-scale internal migration, cancer registration based on HRP may introduce systematic selection bias by failing to capture cases occurring among migrant populations, thereby distorting the actual spatial distribution of digestive cancer burden. However, studies that adjust digestive cancer burden for population migration at the district/county level and systematically characterize its spatial distribution and clustering patterns remain scarce. In this study, spatial descriptive analyses based on migration-adjusted estimates revealed pronounced geographic differentiation in the burden of five digestive cancers across China: high-burden areas were predominantly concentrated in eastern coastal, northeastern, and parts of central–eastern provinces, whereas western and parts of southwestern provinces generally exhibited lower levels. This overall pattern is broadly consistent with previous reports of digestive cancer geography in China, while also suggesting that migration adjustment may refine the delineation of high- and low-burden areas (25). Moreover, the spatial locations of high-burden areas tended to form contiguous patterns across neighboring counties rather than appearing as isolated units, indicating that their spatial distribution may not be random. Nevertheless, spatial descriptive alone cannot determine whether such spatial continuity reflects statistically significant spatial clustering, underscoring the need for spatial autocorrelation analyses to further evaluate these patterns.

Global spatial autocorrelation analyses of incidence and mortality for the major digestive cancers showed that, for each cancer, the global Moran’s *I* values were significantly positive among males, females, and the overall population (all *p* < 0.001), indicating significant positive spatial autocorrelation and non-random clustering of high or low values. These differences may reflect variation in the sex composition and health profile of migrant populations across regions. This observation is broadly consistent with previous studies suggesting that internal migration in China is selective by sex and health status, which may contribute to sex-specific differences in regional disease burden (26). In 2016, males accounted for a disproportionately large share of the migrant population (52.1%), which may correspond to the more concentrated case distributions observed in certain regions. Moreover, males generally exhibit higher risks of digestive cancers and are more prone to spatial clustering in specific areas, further amplifying the manifestation of high–high clusters within the male population (26–28). In addition to HH and LL clusters, HL and LH areas were also identified. Given their limited interpretability, these patterns were not the focus of the present study but may provide reference for future targeted investigations.

After characterizing sex-specific HH and LL clustering patterns as well as anomalous clusters (HL and LH), we further examined cancer-specific spatial clustering patterns of migration-adjusted incidence and mortality for different digestive cancers. The HH and LL clusters identified by local spatial autocorrelation analysis closely corresponded to the geographic patterns observed in the thematic maps. Overall, high-burden clusters were mainly concentrated in southeastern and coastal areas for liver cancer, eastern and northeastern regions for colorectal cancer, eastern and parts of northwestern China for gastric cancer, central-to-eastern regions for esophageal cancer, and eastern coastal and northeastern regions for pancreatic cancer. These patterns are broadly consistent with previous studies reporting marked geographic heterogeneity in digestive cancers across China (7, 8, 29). However, unlike analyses based on HRP registry statistics, the migration-adjusted estimates used in this study explicitly account for population mobility and may therefore provide an alternative perspective on local disease burden and clustering patterns in the context of population mobility.

Meanwhile, LL clusters of five digestive cancers exhibited relatively well-defined spatial patterns, being predominantly distributed in western, parts of central-western, and southwestern regions, and closely corresponding to areas with low incidence. These LL clusters likely represent not only geographically contiguous low-burden areas, but also broader regional contrasts in the determinants of digestive cancer burden. Such contrasts may be related to the combined influence of population mobility, demographic composition, regional risk exposure, and healthcare conditions (30–32). In this context, migration adjustment may be particularly informative because it helps distinguish areas with persistently low burden from those whose apparent burden may be influenced by population mobility. However, these possible explanations should be interpreted with caution, and the relative role of each factor remains to be further clarified. From a public health perspective, HH clusters should be prioritized in digestive cancer prevention and control efforts, whereas LL clusters provide an important reference for understanding the structural characteristics of regions with relatively low cancer burden.

The spatial distribution of migration-adjusted mortality for five digestive cancers in China was largely consistent with that of incidence, likewise exhibiting pronounced cancer-specific clustering patterns. HH clusters were predominantly distributed in eastern and northeastern provinces, whereas LL clusters were mainly concentrated in western and parts of central-western provinces. The spatial locations of mortality HH and LL clusters largely overlapped with those identified for incidence. This pattern suggests that the spatial differentiation of mortality may be associated with multiple regional factors in addition to migration-adjusted incidence itself. In the context of population migration, possible explanations include differences in demographic composition, health profiles, and healthcare conditions between migrant-receiving and migrant-sending regions. For example, working-age adults constitute a relatively large share of migrant populations, which may partly relate to lower observed mortality in some migrant-receiving areas. In addition, disparities in healthcare accessibility and quality may also contribute to the regional clustering of mortality (33–35). Taken together, these factors may jointly help explain the observed mortality clustering; however, these interpretations remain speculative and were not directly tested in the present study. Their specific contributions therefore require further investigation.

From a prevention and control perspective, these findings provide a spatial foundation for developing targeted intervention strategies by integrating population mobility characteristic. Because population mobility may distort burden estimates based on HRP, migration-adjusted spatial clustering may provide a more relevant basis for regional prioritization in cancer prevention and control. By jointly considering HH and LL patterns of incidence and mortality, differentiated interventions may be aligned with different regional profiles. Areas with HH incidence may warrant strengthened early screening and health education to improve cancer detection and prevention, whereas areas with HH mortality may require greater attention to standardized diagnosis and treatment, as well as treatment accessibility, in order to improve outcomes and survival. Areas with LL incidence may serve as useful reference regions for understanding structural characteristics associated with relatively low burden, while continued surveillance remains important for monitoring changes in risk and population mobility. Similarly, areas with LL mortality may provide useful insight into regional conditions associated with relatively low fatality burden and may inform efforts to maintain adequate healthcare coverage and early diagnostic services.

Beyond its specific findings, this study contributes to the literature in both methodological and practical terms. Methodologically, by integrating migration-adjusted burden estimates with spatial description and spatial autocorrelation analysis at the district/county level, this study provides a more informative perspective for understanding the spatial distribution of digestive cancer burden in the context of large-scale population mobility. Practically, the identification of HH and LL clusters of incidence and mortality offers a useful analytical framework for regional prioritization in cancer prevention and control, provides a reference for the rational allocation of public health resources, and may also provide a basis for regionally targeted prevention and control strategies. Given its scalability, this framework may also provide useful reference for related spatial studies in other countries or regions similarly affected by substantial population mobility.

This study has several limitations. First, the migration-adjusted estimates were based on data from 2016, and their applicability to other years should therefore be interpreted with caution. In addition, the underlying cancer registry data were not evenly distributed across regions, and some areas had relatively sparse registry coverage (for example, only one registry point in Xizang), so area-specific findings in those regions should be interpreted more cautiously. Second, due to data availability constraints, population mobility was characterized using net migrant population rather than more detailed migration measures, which may limit the precision of the adjustment. Third, spatial autocorrelation analysis identifies statistical associations rather than causal relationships; thus, the observed spatial patterns of cancer burden should be interpreted with caution regarding their underlying mechanisms. In addition, Local Moran’s *I* analysis involves multiple area-level comparisons. Overall, the spatial patterns reported in this study should be understood as reflecting the best available evidence for 2016, and future studies should further validate these findings using more recent data and more detailed migration measures, while also assessing the robustness of the identified local clustering patterns.

In conclusion, this study identified significant spatial clustering in the migration-adjusted burden of the five major digestive cancers in China, with largely concordant clustering patterns for incidence and mortality. High-burden clusters of liver and colorectal cancers were mainly located in southeastern coastal and northeastern provinces, those of gastric and esophageal cancers were concentrated in central and eastern provinces, and high-burden clusters of pancreatic cancer were primarily observed in eastern coastal, northeastern, and parts of northwestern provinces. These findings provide spatial epidemiological evidence to inform region-specific cancer prevention and control strategies.

## Data Availability

The original contributions presented in the study are included in the article/Supplementary material, further inquiries can be directed to the corresponding authors.

## References

[1] Cao W Qin K Li F Chen W. Comparative study of cancer profiles between 2020 and 2022 using global cancer statistics (GLOBOCAN). J Natl Cancer Cent. (2024) 4:128–34. doi: 10.1016/j.jncc.2024.05.001, 39282581 PMC11390618

[2] International Agency for Research on Cancer (IARC). Cancer today: estimated cancer incidence and mortality data. Global Cancer Observatory. (2022) Available online at: https://gco.iarc.who.int/today/en (Accessed January 10, 2026).

[3] Han B Zheng R Zeng H Wang S Sun K Chen R . Cancer incidence and mortality in China, 2022. J Natl Cancer Cent. (2024) 4:47–53. doi: 10.1016/j.jncc.2024.01.006, 39036382 PMC11256708

[4] Danpanichkul P Suparan K Tothanarungroj P Dejvajara D Rakwong K Pang Y . Epidemiology of gastrointestinal cancers: a systematic analysis from the global burden of disease study 2021. Gut. (2024) 74:26–34. doi: 10.1136/gutjnl-2024-333227, 39242191

[5] Wang S Zheng R Li J Zeng H Li L Chen R . Global, regional, and national lifetime risks of developing and dying from gastrointestinal cancers in 185 countries: a population-based systematic analysis of GLOBOCAN. Lancet Gastroenterol Hepatol. (2024) 9:229–37. doi: 10.1016/S2468-1253(23)00366-7, 38185129 PMC10849975

[6] Xu J Gong J Han H Wang Z Wang W Wang L . Co-occurrence patterns of esophageal and stomach cancer across 204 countries and territories: a spatial correspondence and systematic analysis. Front Oncol. (2025) 15:1613839. doi: 10.3389/fonc.2025.1613839, 41179660 PMC12576481

[7] Li P Qi X Bai R Yang M Jing J Xia R . The spatiotemporal associations between esophageal and gastric cancers provide evidence for its joint endoscopic screening in China: a population-based study. BMC Med. (2024) 22:364. doi: 10.1186/s12916-024-03594-7, 39232729 PMC11375892

[8] Zhang L Wan X Shi R Gong P Si Y. Comparing spatial patterns of 11 common cancers in mainland China. BMC Public Health. (2022) 22:1551. doi: 10.1186/s12889-022-13926-y, 35971087 PMC9377081

[9] Liao Y Li C Xia C Zheng R Xu B Zeng H . Spatial distribution of esophageal cancer mortality in China: a machine learning approach. Int Health. (2021) 13:70–9. doi: 10.1093/inthealth/ihaa022, 32478387 PMC7807241

[10] Jiang F Fu Z Lu Z Chu J Xu A Guo X . Cancer survival analysis and spatial distribution during 2014-2016 in Shandong Province, China. Sci Rep. (2023) 13:10324. doi: 10.1038/s41598-023-37252-4, 37365230 PMC10293178

[11] National Bureau of Statistics of China. Main data of the seventh national population census. (2021) Available online at: http://www.stats.gov.cn/english/PressRelease/202105/t20210510_1817185.html (Accessed January 10, 2026).

[12] Wei W Zeng H Zheng R Zhang S An L Chen R . Cancer registration in China and its role in cancer prevention and control. Lancet Oncol. (2020) 21:e342–9. doi: 10.1016/S1470-2045(20)30073-5, 32615118

[13] Hao S Li G Sun H Du L Zhang Y Wang X . Migration-adjusted lung cancer burden in China: a population data-based Bayesian spatial modeling approach. JNCI Cancer Spectr. (2026) 10:pkag027. doi: 10.1093/jncics/pkag027, 41834133 PMC13061136

[14] World Health Organization (WHO). International statistical classification of diseases and related health problems, 10th revision (ICD-10). (2019) Available online at: https://icd.who.int/browse10/2019/en (Accessed January 11, 2026).

[15] National Cancer Center. China cancer Registration Annual Report 2019. Beijing: People’s Medical Publishing House (2021).

[16] Adegboye O Gayawan E James A Adegboye A Elfaki F. Bayesian spatial modelling of Ebola outbreaks in Democratic Republic of Congo through the INLA-SPDE approach. Zoonoses Public Health. (2021) 68:443–51. doi: 10.1111/zph.12828, 33780159

[17] Zhong R Amaral AVR Moraga P. Spatial data fusion adjusting for preferential sampling using integrated nested Laplace approximation and stochastic partial differential equation. J R Stat Soc Ser A Stat Soc. (2025) 188:140–57. doi: 10.1093/jrsssa/qnae058

[18] Teng J Ding S Zhang H Wang K Hu X. Bayesian spatiotemporal modelling analysis of hemorrhagic fever with renal syndrome outbreaks in China using R-INLA. Zoonoses Public Health. (2023) 70:46–57. doi: 10.1111/zph.12999, 36093577

[19] Migrant Population Service Center. The 2016 China migrants dynamic survey technical document. (2016) Available online at: https://www.chinaldrk.org.cn/wjw/#/home (Accessed January 11, 2026).

[20] National Bureau of Statistics of China. China Population & Employment Statistics Yearbook 2016. Beijing: China Statistics Press (2017).

[21] Lin J. Comparison of Moran’s I and Geary’s c in multivariate spatial pattern analysis. Geogr Anal. (2023) 55:685–702. doi: 10.1111/gean.12355

[22] Mahato RK Htike KM Sornlorm K Koro AB Kafle A Sharma V. A spatial autocorrelation analysis of road traffic accidents by severity using Moran's I spatial statistics: a study from Nepal 2019-2022. BMC Public Health. (2024) 24:3086. doi: 10.1186/s12889-024-20586-7, 39511534 PMC11545994

[23] Gedamu WT Plank-Wiedenbeck U Wodajo BT. A spatial autocorrelation analysis of road traffic crash by severity using Moran’s I spatial statistics: a comparative study of Addis Ababa and Berlin cities. Accid Anal Prev. (2024) 200:107535. doi: 10.1016/j.aap.2024.107535, 38489942

[24] Anselin L. Local indicators of spatial association—LISA. Geogr Anal. (1995) 27:93–115. doi: 10.1111/j.1538-4632.1995.tb00338.x

[25] Bian Y Yu X Zhang J Zhao Y Zheng M Tang C . Geographical analysis of malignant tumor incidence and treatment in China. Sci Rep. (2025) 15:32049. doi: 10.1038/s41598-025-17452-w, 40890268 PMC12402056

[26] Tian Y Zhan Y Wu M. Gender differences in migrant workers health in China. Int J Public Health. (2023) 68:1605018. doi: 10.3389/ijph.2023.1605018, 37655264 PMC10467421

[27] Yang X Li R Liu J Shu J Chen H Dong M . Unequal disease burdens: exploring the intersection of gender, health and disease dynamics in China. BMC Public Health. (2025) 25:2182. doi: 10.1186/s12889-025-23354-3, 40604656 PMC12220547

[28] Yang X Zhang Y Zou S Chen Y Cai Z Zhu Y . The role of social integration in chronic disease prevalences among the internal migrant populations in China: evidence from a National Survey. Healthcare. (2025) 13:69. doi: 10.3390/healthcare13010069, 39791676 PMC11719482

[29] Xu L Zhao J Li Z Sun J Lu Y Zhang R . National and subnational incidence, mortality and associated factors of colorectal cancer in China: a systematic analysis and modelling study. J Glob Health. (2023) 13:04096. doi: 10.7189/jogh.13.04096, 37824177 PMC10569376

[30] Kim M Gu H. Relationships between health education, health Behaviors, and health status among migrants in China: a cross-sectional study based on the China migrant dynamic survey. Healthcare. (2023) 11:1768. doi: 10.3390/healthcare11121768, 37372886 PMC10298536

[31] Dong B Zhou Y Wang L Wang Y Zhang Z. Identifying key determinants of health among China’s migrant population using machine learning methods: evidence from the China migrants dynamic survey. PLoS One. (2025) 20:e0335168. doi: 10.1371/journal.pone.0335168, 41212830 PMC12599968

[32] Dong E Xu T Shi J Ba D Zhou H Li Z . Healthy immigration effect among internal migrants in megacities: a cross-sectional study in Shanghai, China. Front Public Health. (2023) 11:1167697. doi: 10.3389/fpubh.2023.1167697, 37377549 PMC10291071

[33] Siqueira TS Silva JRS Souza MDR Leite DCF Edwards T Martins-Filho PR . Spatial clusters, social determinants of health and risk of maternal mortality by COVID-19 in Brazil: a national population-based ecological study. Lancet Reg Health Am. (2021) 3:100076. doi: 10.1016/j.lana.2021.100076, 34541570 PMC8432892

[34] Yang X Chan KW. Forever young: China’s migration regime and age patterns. Eurasian Geogr Econ. (2025) 66:469–96. doi: 10.1080/15387216.2023.2279545, 40740544 PMC12308837

[35] Zhai X Zhou Z Lai S Wang J Zhao Y Liu G . Decomposing disparities in the utilization of basic public health services between locals and internal migrants in China: the role of social determinants. Int J Equity Health. (2025) 24:9. doi: 10.1186/s12939-024-02371-5, 39794815 PMC11724435

