# Supplementary material for: Integrating migration into cancer geography: spatial clustering of five digestive cancer burden in China
Source: Front Public Health. 2026 May 22;14:1791882. doi: 10.3389/fpubh.2026.1791882 (PMC13236963; doi:10.3389/fpubh.2026.1791882)
Supplement: Supplementary file 1 [file Data_Sheet_1.pdf]

Supplementary Material

1 Supplementary Tables

Table S1. Proportion of districts/counties classified as HH clusters based on Local Moran's *I* for incidence of liver, colorectal, and gastric cancers by province in China

Province	Liver cancer			Colorectal cancer			Gastric cancer		
	Male	Female	All	Male	Female	All	Male	Female	All
Anhui	0.00 (0/104)	0.16 (17/104)	0.10 (10/104)	0.29 (30/104)	0.31 (32/104)	0.23 (24/104)	0.86 (89/104)	0.83 (86/104)	0.79 (82/104)
Beijing	0.00 (0/16)	0.00 (0/16)	0.00 (0/16)	0.00 (0/16)	0.00 (0/16)	0.00 (0/16)	0.00 (0/16)	0.00 (0/16)	0.00 (0/16)
Chongqing	0.61 (23/38)	0.29 (11/38)	0.53 (20/38)	0.16 (6/38)	0.16 (6/38)	0.11 (4/38)	0.00 (0/38)	0.00 (0/38)	0.00 (0/38)
Fujian	0.77 (65/84)	0.21 (18/84)	0.74 (62/84)	0.80 (67/84)	0.75 (63/84)	0.85 (71/84)	0.62 (52/84)	0.57 (48/84)	0.67 (56/84)
Gansu	0.01 (1/86)	0.62 (53/86)	0.03 (3/86)	0.00 (0/86)	0.00 (0/86)	0.00 (0/86)	0.90 (77/86)	0.90 (77/86)	0.72 (62/86)
Guangdong	0.68 (84/124)	0.07 (9/124)	0.59 (73/124)	0.73 (91/124)	0.59 (73/124)	0.80 (99/124)	0.00 (0/124)	0.00 (0/124)	0.00 (0/124)
Guangxi	0.89 (99/111)	0.60 (67/111)	0.86 (95/111)	0.26 (29/111)	0.43 (48/111)	0.16 (18/111)	0.00 (0/111)	0.00 (0/111)	0.00 (0/111)
Guizhou	0.50 (44/88)	0.80 (70/88)	0.52 (46/88)	0.55 (48/88)	0.63 (55/88)	0.36 (32/88)	0.00 (0/88)	0.00 (0/88)	0.00 (0/88)
Hainan	0.96 (23/24)	0.00 (0/24)	0.96 (23/24)	0.58 (14/24)	0.54 (13/24)	0.58 (14/24)	0.00 (0/24)	0.00 (0/24)	0.00 (0/24)
Hebei	0.00 (0/167)	0.00 (0/167)	0.00 (0/167)	0.02 (3/167)	0.02 (3/167)	0.01 (2/167)	0.29 (49/167)	0.29 (49/167)	0.28 (47/167)
Heilongjiang	0.24 (29/121)	0.23 (28/121)	0.23 (28/121)	0.73 (88/121)	0.79 (95/121)	0.60 (73/121)	0.02 (2/121)	0.02 (2/121)	0.00 (0/121)
Henan	0.00 (0/157)	0.36 (57/157)	0.08 (12/157)	0.00 (0/157)	0.00 (0/157)	0.00 (0/157)	0.74 (116/157)	0.70 (110/157)	0.76 (120/157)
Hubei	0.07 (7/103)	0.20 (21/103)	0.05 (5/103)	0.00 (0/103)	0.00 (0/103)	0.01 (1/103)	0.17 (18/103)	0.17 (17/103)	0.23 (24/103)
Hunan	0.18 (22/122)	0.11 (14/122)	0.19 (23/122)	0.02 (3/122)	0.04 (5/122)	0.00 (0/122)	0.00 (0/122)	0.00 (0/122)	0.00 (0/122)

Inner Mongolia	0.18 (19/103)	0.00 (0/103)	0.16 (16/103)	0.11 (11/103)	0.17 (17/103)	0.06 (6/103)	0.03 (3/103)	0.03 (3/103)	0.01 (1/103)
Jiangsu	0.00 (0/95)	0.06 (6/95)	0.00 (0/95)	0.66 (63/95)	0.64 (61/95)	0.68 (65/95)	0.93 (88/95)	0.88 (84/95)	0.93 (88/95)
Jiangxi	0.19 (19/100)	0.01 (1/100)	0.16 (16/100)	0.13 (13/100)	0.09 (9/100)	0.19 (19/100)	0.26 (26/100)	0.18 (18/100)	0.42 (42/100)
Jilin	0.28 (17/60)	0.48 (29/60)	0.33 (20/60)	0.60 (36/60)	0.67 (40/60)	0.45 (27/60)	0.00 (0/60)	0.00 (0/60)	0.00 (0/60)
Liaoning	0.27 (27/100)	0.03 (3/100)	0.18 (18/100)	0.96 (96/100)	0.95 (95/100)	0.86 (86/100)	0.08 (8/100)	0.08 (8/100)	0.08 (8/100)
Ningxia	0.00 (0/22)	0.32 (7/22)	0.00 (0/22)	0.00 (0/22)	0.00 (0/22)	0.00 (0/22)	0.45 (10/22)	0.59 (13/22)	0.36 (8/22)
Qinghai	0.24 (11/45)	0.60 (27/45)	0.36 (16/45)	0.02 (1/45)	0.02 (1/45)	0.00 (0/45)	0.67 (30/45)	0.76 (34/45)	0.60 (27/45)
Shaanxi	0.00 (0/107)	0.03 (3/107)	0.01 (1/107)	0.00 (0/107)	0.00 (0/107)	0.00 (0/107)	0.42 (45/107)	0.55 (59/107)	0.15 (16/107)
Shandong	0.08 (11/136)	0.01 (2/136)	0.04 (5/136)	0.11 (15/136)	0.13 (18/136)	0.10 (14/136)	0.68 (93/136)	0.74 (101/136)	0.54 (73/136)
Shanghai	0.00 (0/16)	0.00 (0/16)	0.00 (0/16)	1.00 (16/16)	1.00 (16/16)	1.00 (16/16)	0.69 (11/16)	0.69 (11/16)	0.81 (13/16)
Shanxi	0.00 (0/117)	0.00 (0/117)	0.00 (0/117)	0.00 (0/117)	0.00 (0/117)	0.00 (0/117)	0.60 (70/117)	0.61 (71/117)	0.60 (70/117)
Sichuan	0.20 (36/183)	0.03 (6/183)	0.16 (29/183)	0.17 (32/183)	0.22 (40/183)	0.12 (22/183)	0.14 (25/183)	0.15 (28/183)	0.10 (19/183)
Tianjin	0.00 (0/16)	0.00 (0/16)	0.00 (0/16)	0.00 (0/16)	0.00 (0/16)	0.00 (0/16)	0.00 (0/16)	0.00 (0/16)	0.00 (0/16)
Xinjiang	0.35 (37/107)	0.00 (0/107)	0.30 (32/107)	0.03 (3/107)	0.04 (4/107)	0.01 (1/107)	0.00 (0/107)	0.00 (0/107)	0.00 (0/107)
Xizang	0.16 (12/74)	0.86 (64/74)	0.43 (32/74)	0.00 (0/74)	0.00 (0/74)	0.00 (0/74)	0.00 (0/74)	0.00 (0/74)	0.00 (0/74)
Yunnan	0.03 (4/129)	0.03 (4/129)	0.03 (4/129)	0.03 (4/129)	0.02 (2/129)	0.18 (23/129)	0.00 (0/129)	0.00 (0/129)	0.00 (0/129)
Zhejiang	0.39 (35/90)	0.13 (12/90)	0.36 (32/90)	0.98 (88/90)	0.96 (86/90)	0.96 (86/90)	0.78 (70/90)	0.78 (70/90)	0.79 (71/90)

Note: For each cancer type, the value represents the proportion of districts/counties within a province that were identified as HH clusters based on Local Moran's *I* analysis of incidences. The values in parentheses indicate the number of HH cluster districts/counties over the total number of districts/counties in that province. Higher values indicate a greater spatial concentration of high-risk areas within the province..

Table S1 (continued). Proportion of districts/counties classified as HH clusters based on Local Moran's *I* for incidence of esophageal and pancreatic cancers by province in China

Province	Esophageal cancer			Pancreatic cancer		
	Male	Female	All	Male	Female	All
Anhui	0.79 (82/104)	0.82 (85/104)	0.71 (74/104)	0.46 (48/104)	0.49 (51/104)	0.44 (46/104)
Beijing	0.00 (0/16)	0.00 (0/16)	0.00 (0/16)	0.00 (0/16)	0.13 (2/16)	0.00 (0/16)
Chongqing	0.50 (19/38)	0.55 (21/38)	0.42 (16/38)	0.00 (0/38)	0.00 (0/38)	0.00 (0/38)
Fujian	0.26 (22/84)	0.35 (29/84)	0.00 (0/84)	0.01 (1/84)	0.02 (2/84)	0.00 (0/84)
Gansu	0.21 (18/86)	0.23 (20/86)	0.15 (13/86)	0.14 (12/86)	0.01 (1/86)	0.42 (36/86)
Guangdong	0.07 (9/124)	0.12 (15/124)	0.00 (0/124)	0.00 (0/124)	0.00 (0/124)	0.00 (0/124)
Guangxi	0.00 (0/111)	0.00 (0/111)	0.00 (0/111)	0.00 (0/111)	0.00 (0/111)	0.00 (0/111)
Guizhou	0.00 (0/88)	0.00 (0/88)	0.00 (0/88)	0.00 (0/88)	0.00 (0/88)	0.00 (0/88)
Hainan	0.00 (0/24)	0.00 (0/24)	0.00 (0/24)	0.00 (0/24)	0.00 (0/24)	0.00 (0/24)
Hebei	0.34 (56/167)	0.30 (50/167)	0.38 (63/167)	0.12 (20/167)	0.16 (26/167)	0.11 (19/167)
Heilongjiang	0.00 (0/121)	0.00 (0/121)	0.00 (0/121)	0.93 (113/121)	0.95 (115/121)	0.90 (109/121)
Henan	0.93 (146/157)	0.87 (136/157)	0.99 (155/157)	0.00 (0/157)	0.00 (0/157)	0.00 (0/157)
Hubei	0.19 (20/103)	0.22 (23/103)	0.15 (15/103)	0.00 (0/103)	0.00 (0/103)	0.00 (0/103)
Hunan	0.00 (0/122)	0.00 (0/122)	0.00 (0/122)	0.00 (0/122)	0.00 (0/122)	0.00 (0/122)
Inner Mongolia	0.02 (2/103)	0.12 (12/103)	0.01 (1/103)	0.60 (62/103)	0.56 (58/103)	0.59 (61/103)
Jiangsu	0.86 (82/95)	0.86 (82/95)	0.87 (83/95)	0.88 (84/95)	0.88 (84/95)	0.87 (83/95)
Jiangxi	0.00 (0/100)	0.00 (0/100)	0.00 (0/100)	0.00 (0/100)	0.00 (0/100)	0.00 (0/100)
Jilin	0.00 (0/60)	0.02 (1/60)	0.00 (0/60)	0.98 (59/60)	0.98 (59/60)	0.92 (55/60)
Liaoning	0.00 (0/100)	0.03 (3/100)	0.00 (0/100)	1.00 (100/100)	1.00 (100/100)	1.00 (100/100)
Ningxia	0.00 (0/22)	0.00 (0/22)	0.00 (0/22)	0.36 (8/22)	0.00 (0/22)	0.64 (14/22)
Qinghai	0.07 (3/45)	0.07 (3/45)	0.02 (1/45)	0.00 (0/45)	0.00 (0/45)	0.11 (5/45)
Shaanxi	0.57 (61/107)	0.48 (51/107)	0.64 (69/107)	0.00 (0/107)	0.00 (0/107)	0.03 (3/107)
Shandong	0.57 (77/136)	0.59 (80/136)	0.46 (63/136)	0.13 (18/136)	0.13 (18/136)	0.16 (22/136)
Shanghai	0.00 (0/16)	0.00 (0/16)	0.00 (0/16)	1.00 (16/16)	1.00 (16/16)	1.00 (16/16)
Shanxi	0.67 (78/117)	0.57 (67/117)	0.85 (99/117)	0.00 (0/117)	0.00 (0/117)	0.00 (0/117)

Sichuan	0.46<br>(84/183)	0.62<br>(113/183)	0.28<br>(52/183)	0.20<br>(36/183)	0.30<br>(55/183)	0.08<br>(14/183)
Tianjin	0.00<br>(0/16)	0.00<br>(0/16)	0.00<br>(0/16)	0.00<br>(0/16)	0.00<br>(0/16)	0.00<br>(0/16)
Xinjiang	0.00<br>(0/107)	0.00<br>(0/107)	0.00<br>(0/107)	0.00<br>(0/107)	0.00<br>(0/107)	0.00<br>(0/107)
Xizang	0.00<br>(0/74)	0.00<br>(0/74)	0.00<br>(0/74)	0.00<br>(0/74)	0.00<br>(0/74)	0.00<br>(0/74)
Yunnan	0.00<br>(0/129)	0.00<br>(0/129)	0.00<br>(0/129)	0.00<br>(0/129)	0.00<br>(0/129)	0.00<br>(0/129)
Zhejiang	0.00<br>(0/90)	0.00<br>(0/90)	0.00<br>(0/90)	0.88<br>(79/90)	0.90<br>(81/90)	0.84<br>(76/90)

Note: For each cancer type, the value represents the proportion of districts/counties within a province that were identified as HH clusters based on Local Moran's *I* analysis of incidences. The values in parentheses indicate the number of HH cluster districts/counties over the total number of districts/counties in that province. Higher values indicate a greater spatial concentration of high-risk areas within the province.

Table S2. Proportion of districts/counties classified as HH clusters based on Local Moran's *I* for mortality of liver, colorectal, and gastric cancers by province in China

Province	Liver cancer			Colorectal cancer			Gastric cancer		
	Male	Female	All	Male	Female	All	Male	Female	All
Anhui	0.01 (1/104)	0.00 (0/104)	0.15 (16/104)	0.15 (16/104)	0.18 (19/104)	0.11 (11/104)	0.87 (90/104)	0.84 (87/104)	0.88 (92/104)
Beijing	0.00 (0/16)	0.00 (0/16)	0.00 (0/16)	0.00 (0/16)	0.00 (0/16)	0.00 (0/16)	0.00 (0/16)	0.00 (0/16)	0.00 (0/16)
Chongqing	0.66 (25/38)	0.71 (27/38)	0.47 (18/38)	0.42 (16/38)	0.50 (19/38)	0.37 (14/38)	0.00 (0/38)	0.00 (0/38)	0.00 (0/38)
Fujian	0.74 (62/84)	0.80 (67/84)	0.20 (17/84)	0.77 (65/84)	0.80 (67/84)	0.67 (56/84)	0.62 (52/84)	0.58 (49/84)	0.58 (49/84)
Gansu	0.00 (0/86)	0.00 (0/86)	0.60 (52/86)	0.00 (0/86)	0.00 (0/86)	0.00 (0/86)	0.84 (72/86)	0.87 (75/86)	0.77 (66/86)
Guangdong	0.59 (73/124)	0.70 (87/124)	0.07 (9/124)	0.60 (75/124)	0.60 (75/124)	0.60 (75/124)	0.00 (0/124)	0.00 (0/124)	0.00 (0/124)
Guangxi	0.87 (97/111)	0.91 (101/111)	0.56 (62/111)	0.51 (57/111)	0.58 (64/111)	0.39 (43/111)	0.00 (0/111)	0.00 (0/111)	0.00 (0/111)
Guizhou	0.47 (41/88)	0.34 (30/88)	0.65 (57/88)	0.91 (80/88)	0.91 (80/88)	0.91 (80/88)	0.00 (0/88)	0.00 (0/88)	0.00 (0/88)
Hainan	0.96 (23/24)	0.96 (23/24)	0.00 (0/24)	0.00 (0/24)	0.00 (0/24)	0.00 (0/24)	0.00 (0/24)	0.00 (0/24)	0.00 (0/24)
Hebei	0.00 (0/167)	0.00 (0/167)	0.01 (1/167)	0.01 (2/167)	0.02 (4/167)	0.01 (2/167)	0.40 (67/167)	0.40 (67/167)	0.39 (65/167)
Heilongjiang	0.58 (70/121)	0.54 (65/121)	0.70 (85/121)	0.29 (35/121)	0.31 (37/121)	0.22 (27/121)	0.00 (0/121)	0.02 (2/121)	0.00 (0/121)
Henan	0.03 (4/157)	0.00 (0/157)	0.34 (53/157)	0.00 (0/157)	0.00 (0/157)	0.00 (0/157)	0.80 (125/157)	0.75 (117/157)	0.83 (130/157)
Hubei	0.12 (12/103)	0.11 (11/103)	0.27 (28/103)	0.00 (0/103)	0.00 (0/103)	0.01 (1/103)	0.17 (17/103)	0.16 (16/103)	0.23 (24/103)
Hunan	0.11 (14/122)	0.15 (18/122)	0.07 (9/122)	0.21 (26/122)	0.19 (23/122)	0.37 (45/122)	0.00 (0/122)	0.00 (0/122)	0.00 (0/122)
Inner Mongolia	0.16 (16/103)	0.19 (20/103)	0.17 (17/103)	0.05 (5/103)	0.05 (5/103)	0.03 (3/103)	0.01 (1/103)	0.03 (3/103)	0.01 (1/103)
Jiangsu	0.13 (12/95)	0.11 (10/95)	0.18 (17/95)	0.52 (49/95)	0.55 (52/95)	0.51 (48/95)	0.91 (86/95)	0.89 (85/95)	0.88 (84/95)
Jiangxi	0.15 (15/100)	0.20 (20/100)	0.01 (1/100)	0.17 (17/100)	0.17 (17/100)	0.14 (14/100)	0.26 (26/100)	0.15 (15/100)	0.48 (48/100)

Jilin	0.40 (24/60)	0.45 (27/60)	0.47 (28/60)	0.10 (6/60)	0.13 (8/60)	0.05 (3/60)	0.00 (0/60)	0.00 (0/60)	0.00 (0/60)
Liaoning	0.26 (26/100)	0.33 (33/100)	0.19 (19/100)	0.00 (0/100)	0.91 (91/100)	0.92 (92/100)	0.07 (7/100)	0.08 (8/100)	0.02 (2/100)
Ningxia	0.00 (0/22)	0.00 (0/22)	0.27 (6/22)	0.00 (0/22)	0.00 (0/22)	0.00 (0/22)	0.41 (9/22)	0.41 (9/22)	0.41 (9/22)
Qinghai	0.00 (0/45)	0.00 (0/45)	0.33 (15/45)	0.00 (0/45)	0.00 (0/45)	0.00 (0/45)	0.69 (31/45)	0.71 (32/45)	0.58 (26/45)
Shaanxi	0.02 (2/107)	0.01 (1/107)	0.12 (13/107)	0.00 (0/107)	0.00 (0/107)	0.00 (0/107)	0.22 (24/107)	0.27 (29/107)	0.10 (11/107)
Shandong	0.09 (12/136)	0.10 (13/136)	0.10 (13/136)	0.10 (14/136)	0.11 (15/136)	0.10 (14/136)	0.74 (101/136)	0.81 (110/136)	0.69 (94/136)
Shanghai	0.13 (2/16)	0.38 (6/16)	0.38 (6/16)	0.81 (13/16)	0.81 (13/16)	0.81 (13/16)	0.63 (10/16)	0.63 (10/16)	0.56 (9/16)
Shanxi	0.00 (0/117)	0.00 (0/117)	0.00 (0/117)	0.00 (0/117)	0.00 (0/117)	0.00 (0/117)	0.69 (81/117)	0.70 (82/117)	0.67 (78/117)
Sichuan	0.23 (42/183)	0.27 (50/183)	0.27 (50/183)	0.49 (90/183)	0.51 (94/183)	0.38 (70/183)	0.16 (30/183)	0.17 (31/183)	0.14 (25/183)
Tianjin	0.00 (0/16)	0.00 (0/16)	0.00 (0/16)	0.00 (0/16)	0.00 (0/16)	0.00 (0/16)	0.00 (0/16)	0.00 (0/16)	0.00 (0/16)
Xinjiang	0.01 (1/107)	0.00 (0/107)	0.00 (0/107)	0.00 (0/107)	0.00 (0/107)	0.00 (0/107)	0.00 (0/107)	0.00 (0/107)	0.00 (0/107)
Xizang	0.00 (0/74)	0.00 (0/74)	0.00 (0/74)	0.00 (0/74)	0.00 (0/74)	0.00 (0/74)	0.00 (0/74)	0.00 (0/74)	0.00 (0/74)
Yunnan	0.03 (4/129)	0.03 (4/129)	0.03 (4/129)	0.22 (28/129)	0.18 (23/129)	0.38 (49/129)	0.00 (0/129)	0.00 (0/129)	0.00 (0/129)
Zhejiang	0.37 (33/90)	0.43 (39/90)	0.43 (39/90)	0.90 (81/90)	0.93 (84/90)	0.88 (79/90)	0.74 (67/90)	0.76 (68/90)	0.51 (46/90)

Note: For each cancer type, the value represents the proportion of districts/counties within a province that were identified as HH clusters based on Local Moran's *I* analysis of mortalities. The values in parentheses indicate the number of HH cluster districts/counties over the total number of districts/counties in that province. Higher values indicate a greater spatial concentration of high-risk areas within the province..

Table S2. (continued). Proportion of districts/counties classified as HH clusters based on Local Moran's *I* for mortality of esophageal and pancreatic cancers by province in China

Province	Esophageal cancer			Pancreatic cancer		
	Male	Female	All	Male	Female	All
Anhui	0.77 (80/104)	0.75 (78/104)	0.69 (72/104)	0.43 (45/104)	0.41 (43/104)	0.41 (43/104)
Beijing	0.00 (0/16)	0.00 (0/16)	0.00 (0/16)	0.50 (8/16)	0.38 (6/16)	0.50 (8/16)
Chongqing	0.47 (18/38)	0.53 (20/38)	0.42 (16/38)	0.00 (0/38)	0.00 (0/38)	0.00 (0/38)
Fujian	0.36 (30/84)	0.38 (32/84)	0.19 (16/84)	0.06 (5/84)	0.06 (5/84)	0.02 (2/84)
Gansu	0.17 (15/86)	0.17 (15/86)	0.19 (16/86)	0.51 (44/86)	0.35 (30/86)	0.60 (52/86)
Guangdong	0.13 (16/124)	0.15 (18/124)	0.02 (2/124)	0.00 (0/124)	0.00 (0/124)	0.00 (0/124)
Guangxi	0.00 (0/111)	0.00 (0/111)	0.00 (0/111)	0.00 (0/111)	0.00 (0/111)	0.00 (0/111)
Guizhou	0.00 (0/88)	0.00 (0/88)	0.00 (0/88)	0.00 (0/88)	0.00 (0/88)	0.00 (0/88)
Hainan	0.00 (0/24)	0.00 (0/24)	0.00 (0/24)	0.00 (0/24)	0.00 (0/24)	0.00 (0/24)
Hebei	0.22 (36/167)	0.16 (26/167)	0.32 (54/167)	0.16 (27/167)	0.14 (23/167)	0.16 (26/167)
Heilongjiang	0.01 (1/121)	0.02 (2/121)	0.00 (0/121)	0.97 (117/121)	0.98 (118/121)	0.93 (112/121)
Henan	0.94 (147/157)	0.87 (136/157)	0.97 (153/157)	0.00 (0/157)	0.00 (0/157)	0.00 (0/157)
Hubei	0.19 (20/103)	0.21 (22/103)	0.13 (13/103)	0.00 (0/103)	0.00 (0/103)	0.00 (0/103)
Hunan	0.00 (0/122)	0.00 (0/122)	0.00 (0/122)	0.00 (0/122)	0.00 (0/122)	0.00 (0/122)
Inner Mongolia	0.02 (2/103)	0.16 (16/103)	0.01 (1/103)	0.46 (47/103)	0.55 (57/103)	0.38 (39/103)
Jiangsu	0.86 (82/95)	0.88 (84/95)	0.82 (78/95)	0.87 (83/95)	0.89 (85/95)	0.87 (83/95)
Jiangxi	0.00 (0/100)	0.00 (0/100)	0.00 (0/100)	0.00 (0/100)	0.00 (0/100)	0.00 (0/100)
Jilin	0.00 (0/60)	0.00 (0/60)	0.00 (0/60)	0.97 (58/60)	0.97 (58/60)	0.73 (44/60)
Liaoning	0.00 (0/100)	0.15 (15/100)	0.00 (0/100)	1.00 (100/100)	0.99 (99/100)	0.98 (98/100)
Ningxia	0.00 (0/22)	0.00 (0/22)	0.00 (0/22)	0.23 (5/22)	0.00 (0/22)	0.41 (9/22)
Qinghai	0.00 (0/45)	0.00 (0/45)	0.02 (1/45)	0.16 (7/45)	0.07 (3/45)	0.29 (13/45)
Shaanxi	0.33 (35/107)	0.27 (29/107)	0.45 (48/107)	0.00 (0/107)	0.00 (0/107)	0.03 (3/107)
Shandong	0.61 (83/136)	0.64 (87/136)	0.46 (63/136)	0.11 (15/136)	0.12 (16/136)	0.10 (13/136)
Shanghai	0.00 (0/16)	0.00 (0/16)	0.00 (0/16)	1.00 (16/16)	1.00 (16/16)	1.00 (16/16)
Shanxi	0.64 (75/117)	0.48 (56/117)	0.81 (95/117)	0.01 (1/117)	0.03 (3/117)	0.01 (1/117)

Sichuan	0.5 (92/183)	0.63 (115/183)	0.30 (55/183)	0.09 (17/183)	0.21 (38/183)	0.04 (7/183)
Tianjin	0.00 (0/16)	0.00 (0/16)	0.00 (0/16)	0.00 (0/16)	0.00 (0/16)	0.00 (0/16)
Xinjiang	0.00 (0/107)	0.00 (0/107)	0.00 (0/107)	0.16 (17/107)	0.18 (19/107)	0.01 (1/107)
Xizang	0.00 (0/74)	0.00 (0/74)	0.00 (0/74)	0.00 (0/74)	0.00 (0/74)	0.00 (0/74)
Yunnan	0.00 (0/129)	0.02 (2/129)	0.00 (0/129)	0.00 (0/129)	0.00 (0/129)	0.00 (0/129)
Zhejiang	0.03 (3/90)	0.07 (6/90)	0.00 (0/90)	0.97 (87/90)	0.97 (87/90)	0.97 (87/90)

Note: For each cancer type, the value represents the proportion of districts/counties within a province that were identified as high-high HH clusters based on Local Moran's *I* analysis of mortalities. The values in parentheses indicate the number of HH cluster districts/counties over the total number of districts/counties in that province. Higher values indicate a greater spatial concentration of high-risk areas within the province.
